# Error Size Shape Relationships between Motor Variability and Implicit Motor Adaptation

**DOI:** 10.3390/biology12030404

**Published:** 2023-03-03

**Authors:** Naoyoshi Matsuda, Masaki O. Abe

**Affiliations:** 1 Graduate School of Education, Hokkaido University, Sapporo 060-0811, Japan; 2 Faculty of Education, Hokkaido University, Sapporo 060-0811, Japan

**Keywords:** motor variability, motor adaptation, implicit adaptation, motor learning

## Abstract

**Simple Summary:**

This study focused on two characteristics of human motor control regarding an acquired skill. First, when performing a movement and generating errors, our movement is corrected based on the errors even if we do not have the intention to correct them. Second, our movement outcomes are always varied even when trying to perform the same movement repeatedly. Although previous studies have examined the relationship between the magnitude of movement correction and motor variability, their findings have been inconsistent. We hypothesized that the impact of motor variability might depend on the error size driving the movement correction and investigated the relationship between different error sizes. Data showed that larger motor variability led to a greater correction of movement when a large, not small, error occurred. The results provide further knowledge regarding the role that motor variability plays in motor learning.

**Abstract:**

Previous studies have demonstrated the effects of motor variability on motor adaptation. However, their findings have been inconsistent, suggesting that various factors affect the relationship between motor variability and adaptation. This study focused on the size of errors driving motor adaptation as one of the factors and examined the relationship between different error sizes. Thirty-one healthy young adults participated in a visuomotor task in which they made fast-reaching movements toward a target. Motor variability was measured in the baseline phase when a veridical feedback cursor was presented. In the adaptation phase, the feedback cursor was sometimes not reflected in the hand position and deviated from the target by 0°, 3°, 6°, or 12° counterclockwise or clockwise (i.e., error-clamp feedback). Movements during trials following trials with error-clamp feedback were measured to quantify implicit adaptation. Implicit adaptation was driven by errors presented through error-clamp feedback. Moreover, motor variability significantly correlated with implicit adaptation driven by a 12° error. The results suggested that motor variability accelerates implicit adaptation when a larger error occurs. As such a trend was not observed when smaller errors occurred, the relationship between motor variability and motor adaptation might have been affected by the error size driving implicit adaptation.

## 1. Introduction

When repeatedly performing the same action, the outcome is variable. Acquiring motor skills implies performing with reduced motor variability [1], and small motor variability leads to high performance when repeating acquired motor skills under the invariable condition. In contrast, the state of the environment and the body frequently change in our daily lives; hence, we need to adjust motor skills according to the state changes. Interestingly, some previous studies have reported that the rate of motor adaptation is positively related to motor variability before environmental changes [2,3]. However, this finding was not supported by other studies [1,4,5], and some of them reported a negative relationship between the adaptation rate and motor variability [6,7]. This relationship is believed to be task-specific and affected by various factors [8].

Notably, there is a possibility that the effect of motor variability on motor adaptation varies according to the size of the error (i.e., the magnitude of sensory prediction error), which drives motor adaptation, although this has not been discussed. In studies reporting a positive relationship, the perturbation was introduced abruptly [2,3]; in contrast, in studies reporting a negative relationship, visuomotor perturbation was introduced gradually [6,7]. Typically, participants experienced larger errors when introducing the perturbation abruptly and smaller errors when introducing the perturbation gradually. Therefore, we hypothesized that the error size might affect the relationship between motor variability and the rate of motor adaptation.

To examine the relationship between different error sizes, a trial-by-trial adaptation paradigm was utilized for the reaching task in this study [9,10,11], and visual error-clamp feedback was employed instead of standard visuomotor perturbation [12]. Motor adaptation comprises implicit and explicit processes [13,14,15]. Moreover, these two processes have many differences, including neural substrates [12,13,16,17,18,19]. Therefore, the relationship between motor variability and motor adaptation should be examined separately for each process. The error-clamp feedback only allows us to measure implicit processes [12,20]; nevertheless, to the best of our knowledge, it has not been used in previous studies examining the relationship. Using the trial-by-trial adaptation paradigm with error-clamp feedback, we examined the relationship between motor variability and implicit adaptation for different error sizes.

## 2. Materials and Methods

### 2.1. Participants

Thirty-one young adults (12 women, 19 men, age: 21.3 ± 3.0 (mean ± standard deviation (SD))) were recruited in this study. All participants were right-handed, according to the Edinburgh Handedness Inventory [21], and were healthy volunteers who had never been diagnosed with developmental or neurological disorders. This study was approved by the Ethics Committee of the Faculty of Education at Hokkaido University (approval number: 21-08), and written informed consent was obtained from all participants. Nine participants had performed an experimental task similar to the one in this study 2–7 days earlier.

### 2.2. Experimental Task

**Figure 1:**
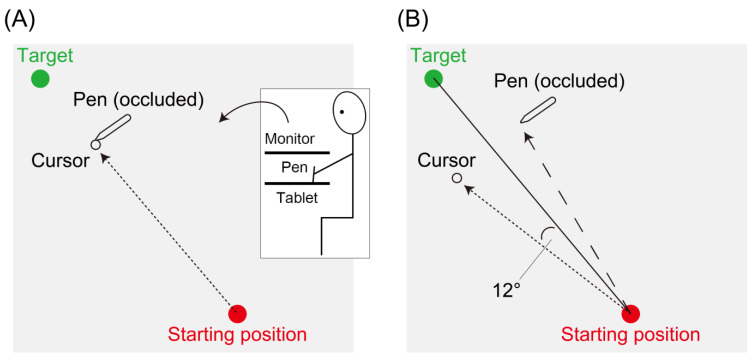
Experimental conditions. (**A**) Veridical feedback. (**B**) Error-clamp feedback. Here, the error-clamp feedback displaying the + 12° error is illustrated.

The participants held a digitizing pen with their right hand and performed reaching movements across a digitizing tablet (Intuos Pro, Wacom, Kazo, Japan) in a darkened room. The pen position was recorded at 120 Hz. All visual stimuli were displayed on a 25-inch LCD monitor (GigaCrysta, I-O DATA DEVICE, Kanazawa, Japan) 28 cm above the tablet. The participants’ direct view of the hand was occluded using a monitor (Figure 1A). Participants moved their hand from a starting position (red circle; 0.6 cm diameter) to a visual target (green circle; 0.6 cm diameter). For each trial, the target was placed 10 cm from the starting position at 115°, 120°, 125°, or 130°, where 0° was to the right of the starting position. The experimental task was controlled via custom software coded in MATLAB (MathWorks, Natick, MA, USA) using Psychtoolbox extensions [22].

The participants had to place their hands on the starting position to initiate each trial. In addition to the starting position, a white ring centered at the starting position was displayed on the monitor. The radius of the ring was the distance between the hand position and the starting position. Guided by the ring, the participants moved their hands back to the starting position. When the radius was within 1 cm, the ring transformed into a visual feedback cursor (white circle; 0.3 cm diameter), indicating the hand position. Once the hand position was maintained within the starting position for 1000 ms, the target appeared. Then, the participants made a fast-reaching movement to the target (i.e., “shooting” movement) without stopping at the target. The visual feedback cursor was provided during the movement; when the movement amplitude exceeded 10 cm, the cursor froze for 1000 ms. If movement time exceeded or fell below the correct time range (100–180 ms), a message “slow” or “fast” was played by the sound system of the computer. Movement time was defined as the interval between the times when the movement amplitude exceeded 1 cm and 10 cm.

### 2.3. Experimental Feedback

There were three types of visual feedback: veridical feedback, no feedback, and error-clamp feedback. In the veridical feedback trial (Figure 1A), the cursor accurately corresponded to the hand position. In the no-feedback trial, the cursor disappeared when the movement amplitude exceeded 1 cm. In the error-clamp trial [12] (Figure 1B), the cursor always deviated from the target at different angles (0°, ±3°, ±6°, and ±12°; error type) regardless of the angular position of the hand, although the movement amplitude was reflected in the distance of the cursor from the starting position. Positive and negative values indicated counterclockwise (CCW) and clockwise (CW) deviations from the target, respectively. Regardless of the types of visual feedback, participants were instructed to move their right hand directly toward the target.

### 2.4. Experimental Protocol

After practicing the shooting movement within the correct time range (Figure S1A,B), the participants performed the baseline and adaptation phases, in that order. The baseline phase had two blocks consisting of 100 trials with veridical feedback (200 trials in total). The order of the target locations was pseudo-randomized so that all four locations (115°, 120°, 125°, and 130°) were presented every four trials. Before the baseline phase, the participants practiced 20 times using the baseline phase procedure.

In the adaptation phase, there were four blocks consisting of 56 trials with veridical feedback, 28 trials with no feedback, and 28 trials with error-clamp feedback (448 trials in total). All error-clamp trials were pseudo-randomly arranged in the adaptation phase so that there were at least two shooting movements under other trial types between each error-clamp trial (Figure S1A). Each error type (i.e., 0°, ±3°, ±6°, and ±12°) was presented four times within a block in random order. Participants could not determine whether the error-clamp feedback would be presented until they began the shooting movement. However, the cursor turned magenta when the movement amplitude exceeded 1 cm in the error-clamp trial. Furthermore, when the amplitude exceeded 10 cm, the cursor remained magenta, and a “knocking” sound was played. Therefore, the participants could understand whether the error-clamp feedback had been introduced after the movement. All of the no-feedback trials were always performed following an error-clamp trial, and the target location in the no-feedback trial was the same as in the last error-clamp trial. Thus, the order of target locations in the adaptation phase was pseudo-randomized so that all four locations were presented every five trials. Before the adaptation phase, participants were fully briefed on error-clamp and no-feedback trials and experienced these trials three times each (Figure S1C). Subsequently, they practiced 20 times using the same procedure as in the adaptation phase, and the phase began. Participants were instructed to move their right hand directly toward the target, regardless of the kind of feedback that the cursor provided. A short break was provided after each block during both phases.

### 2.5. Data Analysis

All analyses were performed using MATLAB software. Trials were precluded when the movement time was more than 1000 ms. We measured the hand angle, defined as the angle between the lines connecting the starting position to the target and the starting position to the hand position when the movement amplitude reached 10 cm. Positive and negative angles indicated CCW and CW deviations from the target, respectively. In the baseline phase, the SD of the hand angle was computed for each participant to quantify the baseline motor variability. In the adaptation phase, error correction was calculated as a measure of implicit adaptation for each participant. Error correction was defined as the hand angle in a no-feedback trial after the error-clamp trial with error (error type: ±3°, ±6°, and ±12°) minus the mean hand angle in no-feedback trials after the error-clamp trial without error (error type: 0°). The sign of the error correction was reversed when the error type was +3°, +6°, and +12°. When calculating the error correction, we excluded no-feedback trials in which the hand angles exceeded the mean ±3 SD of each error type as outliers. All statistical analyses were two-tailed. The significance level was set at α = 0.05, and Bonferroni correction for multiple comparisons was applied when appropriate. This study included participants who had performed an experimental task similar to the one in this study 2–7 days earlier. However, we confirmed that the results were essentially identical when excluding the participants’ data from the analysis.

## 3. Results

**Figure 2:**
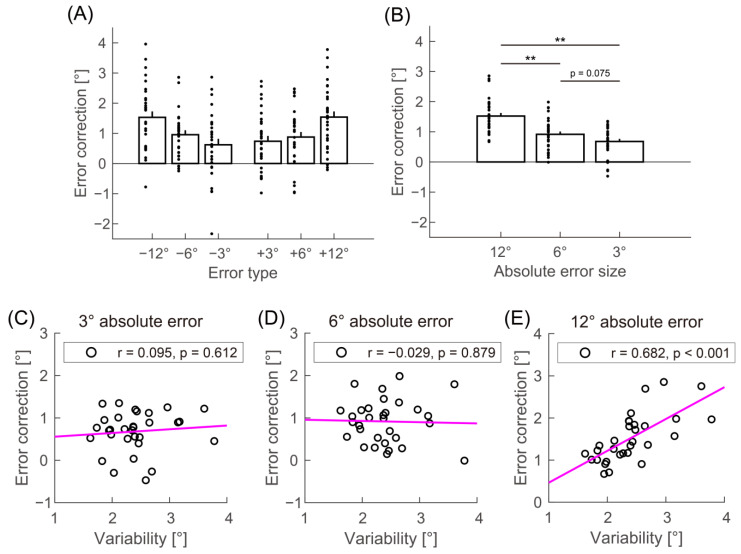
Main results. Error correction for each error type (**A**) and absolute error size (**B**). The error bars represent the standard error of the mean. ** *p* < 0.01. The scatterplots and linear correlation between motor variability and error correction for each absolute error size are as follows: 3° (**C**), 6° (**D**), and 12° absolute error (**E**).

Figure 2 shows that error correction generally had a positive sign for all error types, which is a sign of implicit adaptation. The analysis of variance of the error correction with the error type showed a significant main effect (Figure 2A; F (1.802, 54.051) = 4.887, *p* = 0.014, ηp^2^ = 0.140). In the post hoc analysis conducted following the main effect, the error correction was calculated for each absolute error size, collapsing error direction (i.e., the sign of error). The analysis revealed that error correction was greater for 12° than for 3° and 6° absolute error (Figure 2B; 3° vs. 6°: t (30) = 2.298, p_adj_ = 0.075, d = 0.452; 3° vs. 12°: t (30) = 8.166, p_adj_ < 0.001, d = 1.606; 6° vs. 12°: t (30) = 5.867, p_adj_ < 0.001, d = 1.154).

The primary purpose of this study was to examine the relationship between motor variability and implicit adaptation. The error correction was calculated for each absolute error size as in the above post hoc analysis because we focused on the relationship per error size. Pearson correlation analysis revealed that the SD of the hand angle in the baseline phase was significantly correlated with the error correction for 12° absolute error (Figure 2C; r = 0.682, *p* < 0.001) but not for the other sizes of error (Figure 2D,E; 3°: r = 0.095, *p* = 0.612; 6°: r = −0.029, *p* = 0.879). Here, the Bonferroni corrected level of significance was 0.017 (corrected for the three comparisons). The results indicated that participants who exhibited greater variability in the baseline phase adapted more significantly to a 12° absolute error.

**Figure 3:**
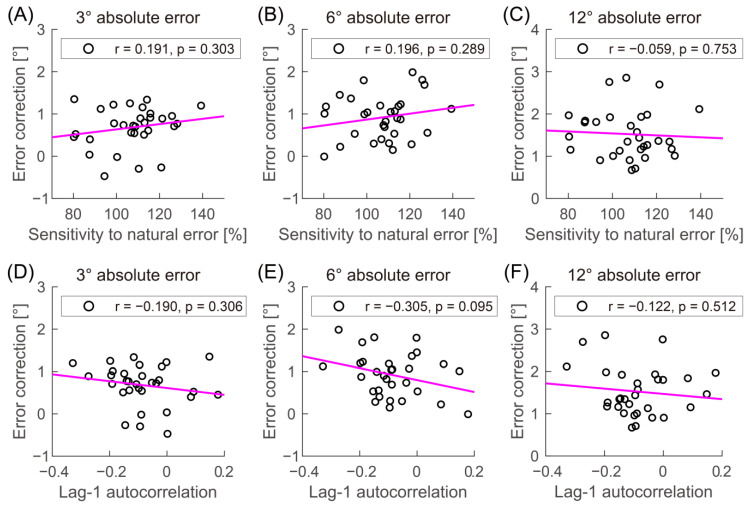
Relationship between error correction and other measurements in the baseline phase. The scatterplot and linear correlation between error correction for each absolute error size and the sensitivity to a natural error (**A**–**C**) and lag-1 autocorrelation (**D**–**F**) are illustrated.

Motor variability in the baseline phase might reflect sensitivity to errors that occur under natural conditions without visuomotor perturbation; for example, overcorrecting the errors could lead to greater variability. Therefore, our results might be explained by the trend that the more excessive the correction of errors in the baseline phase, the higher the error correction in the adaptation phase. To verify this, we calculated the sensitivity to a natural error defined by the fraction of the hand angle at trial N corrected at trial N + 1 (i.e., $\left[\frac{\left(Hand\;angle\;at\;trial\;N\right)-\left(Hand\;angle\;at\;trial\;N+1\right)}{\left(Hand\;angle\;at\;trial\;N\right)}\right]\times 100$; 107.2% ± 14.7%). The denominator, “Hand angle at trial N”, indicates the angular error at trial N since this study defined hand angle as the hand position relative to the target. Furthermore, we calculated the lag-1 autocorrelation in the baseline phase; a negative lag-1 autocorrelation indicates the overcorrection of errors (−0.085 ± 0.112). The first five trials in each block of the baseline phase were excluded from the analysis to avoid the influence of the correction for large initial errors [23]. Pearson correlation analysis revealed that the error correction for any error size was not significantly correlated with the sensitivity to natural error (all −0.059 < r < 0.196, *p* > 0.289; Figure 3A–C) and lag-1 autocorrelation (all −0.305 < r < −0.122, *p* > 0.095; Figure 3D–F). The results suggest that sensitivity did not explain the relationship between motor variability and implicit adaptation driven by the absolute 12° error.

**Figure 4:**
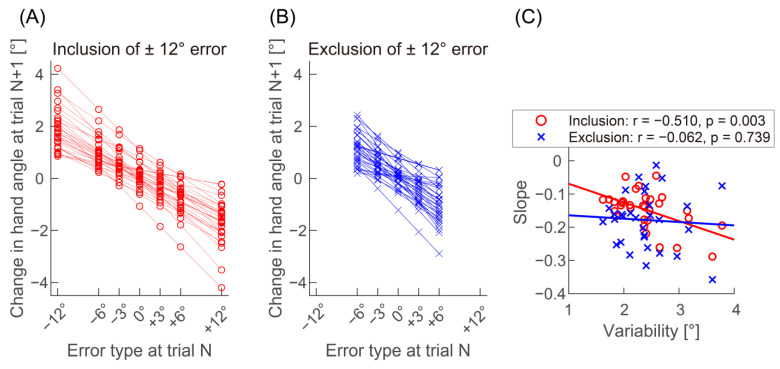
Linear slope of the change in hand angle against the error type. This study calculated changes in hand angle from an error-clamp trial (N) to the subsequent no-feedback trial (N + 1) for each participant. Data from ± 12° error are included in (**A**) and excluded in (**B**). (**C**) illustrates the scatterplot and linear correlation between the slope and motor variability.

In several studies using a trial-by-trial adaptation paradigm, a linear function was fitted to the effect of the preceding perturbation or error on the hand trajectory as a function of the perturbation or error size, and the adaptation rate was quantified by the slope of the fitted linear function [8,24,25,26,27]. Following previous research, as an explorative analysis, we computed changes in hand angle from an error-clamp trial (N) to the subsequent no-feedback trial (N + 1) for each participant [24,27,28] and investigated the correlation of the baseline variability with the linear slope of the change in hand angle against the error type at trial N. The slopes were negative for all participants (Figure 4A; −0.148 ± 0.057); a more negative slope indicates a higher adaptation rate. According to Pearson correlation analysis, the slope was significantly and negatively related with motor variability (r = −0.510, *p* = 0.003). However, our results (Figure 2) predicted that the significant correlation would be attributed to the data from a ± 12° error. In fact, when excluding the data from the slope calculation (Figure 4B; −0.178 ± 0.084), no significant correlation was found (Figure 4C; r = −0.062, *p* = 0.739).

## 4. Discussion

This study has two unique features compared with previous studies that have examined the relationship between motor variability and motor adaptation: a focus on the size of errors driving motor adaptation and the implicit process of motor adaptation. This study showed that motor variability was not significantly related to implicit adaptation driven by a 3° and 6° absolute error, generated through error-clamp feedback. A 3° error was small enough to often occur even during the baseline phase, in which only veridical feedback was presented (absolute hand angle during the baseline phase: 1.9° ± 0.4°; Figure S2). Thus, the results would be inconsistent with some studies showing a negative relationship between motor variability and the rate of motor adaptation to gradual changes in visuomotor perturbation, which frequently causes small errors [6,7]. As the cause of the inconsistency, we thought of three factors. The first was environmental consistency. The environment is relatively stable in reaching tasks that change the perturbation gradually, whereas the environment was unstable during the adaptation phase in our study, where various feedbacks were presented. The rate of motor adaptation is modulated by the consistency of the environment (i.e., visuomotor perturbation) [24,25,29]. Through the modulation of motor adaptation, the relationship between motor variability and implicit adaptation may vary according to environmental consistency. The second factor was the effect of the explicit process of motor adaptation. Although the gradual perturbation method is assumed to mainly measure implicit processes, the method does not eliminate the explicit process as much as the error-clamp method that this study employed does. Participants could explicitly change their aim when they noticed the presence of perturbation or when they noticed a tendency for errors to occur in the direction of the perturbation, even if they did not notice the perturbation itself. If so, participants with a small variability might easily notice the perturbation or tendency and perform re-aiming frequently. As a result, they could adapt to gradual changes in perturbation at a higher rate. Third, in this study, the magnitude of motor adaptation driven by the 3° and 6° absolute errors may have no room for interindividual variation. Specifically, the small errors may not have driven motor adaptation sufficiently enough to examine the individual differences, resulting in a floor effect. Consequently, this study may have found no significant relationship between motor variability and error correction for the 3° and 6° absolute errors.

This study showed that motor variability was significantly positively related to error correction for a 12° absolute error, which was so large that it rarely occurred in the baseline phase. This result is in line with some studies showing a positive relationship between motor variability and the rate of motor adaptation to an abrupt change in visuomotor perturbation, which would cause large errors [2,3]. It has been assumed that motor variability also reflects explicit exploration for better motor plans under the current state of the environment and body, and that the exploration would benefit motor learning [30,31]. However, we measured the implicit process of motor adaptation; thus, this study implies that explicit as well as implicit processes contribute to the positive relationship of motor adaptation with motor variability.

Recently, the proprioceptive re-alignment model (PReMo) has been developed to explain implicit motor adaptation [32]. Visuo-proprioceptive discrepancy caused by visuomotor perturbation and error-clamp feedback paradigms generates the shift in the perceived hand position toward the visual cursor (i.e., proprioceptive shift) [13,33,34,35,36]. PReMo argues that the proprioceptive shift produces a proprioceptive error, the mismatch between the perceived hand position and its intended goal, and motor adaptation can be framed as minimizing the proprioceptive error (see [32] for more detail). Notably, PReMo may predict the positive relationship between the magnitude of motor variability and trial-by-trial adaptation (cf., Appendix A). According to the model, the high variability increases the proprioceptive error, which drives trial-by-trial adaptation and is positively correlated with its magnitude. However, the model also assumes that motor variability does not affect the proprioceptive error when the cursor deviates too far from the target. The current study demonstrates a significant relationship between motor variability and implicit motor adaptation driven by a 12° absolute error; thus, results might not be consistent with PReMo. However, we should also note the potential floor effect that would cause no significant correlation regarding the 3° and 6° absolute errors, as described above.

Although speculative, our results can be explained by a relevance estimation model that proposes that the sensorimotor system estimates the relevance of perceived errors [9]. According to the model, small errors are attributed to miscalibration in the sensorimotor system (relevant factors), and the adaptation rate increases; conversely, large errors are attributed to external sources (irrelevant factors), and the adaptation rate decreases. We think that the magnitude of motor variability may affect the relevance estimation. High motor variability would provide more opportunities to experience larger errors compared to low motor variability. Therefore, when a large error occurs, the ease of associating the error with self-movement may vary according to the magnitude of motor variability. In our study, participants with large variability may have been less unlikely to regard the 12° absolute error as irrelevant compared to participants with small variability (Figure S2); consequently, participants with large variability may have greatly modified their shooting movements based on the error. However, in addition to the interpretation being too hypothetical at present, an insignificant relationship between motor variability and the rate of motor adaptation was also reported in previous studies that introduced abrupt visuomotor perturbation [8,37]. Thus, our interpretation using the relevance estimation model is not applicable to previous findings.

It should be noted that multiple factors would affect the relationship between motor variability and the rate of motor adaptation, as previous studies have suggested [4,8]. One of the factors that they focused on was the source of the motor variability. Motor variability is often divided into planning noise and execution noise [7,8,23,38], and it has been suggested that the rate of motor adaptation is accelerated by planning noise and decelerated by execution noise [7,8]. As a limitation, our study did not measure motor variability by the sources separately. Furthermore, we have two concerns about this study. The first relates to the small sample size. A recent review article raised small sample size as a problem with studies examining individual differences in motor learning [39]. The small sample size not only decreases statistical power but also increases the possibility of generating spurious high correlations. Second, it may be difficult to quickly switch explicit strategies according to the cursor types (i.e., veridical and error-clamp feedback) in our experimental task, as pointed out by one of our reviewers. During the adaptation phase, the veridical feedback should be explicitly utilized in the following trial to adjust participants’ movements, but the error-clamp feedback should not. However, because participants could not determine whether the error-clamp feedback would be presented until they began the shooting movement (see Section 2.4), there may have been trials in which they failed to switch the strategies. In future studies, in addition to overcoming these concerns, we should address the following: (1) revealing the relationship between each noise (i.e., planning and execution noises) and implicit motor adaptation driven by different error sizes and (2) further examining whether it is reasonable to explain the relationship between motor variability and implicit motor adaptation by PReMo or the relevance estimation model.

## 5. Conclusions

In conclusion, this study observed a significant positive relationship between motor variability and implicit adaptation driven by a 12° absolute error but not a 3° or 6° absolute error. The results indicated that greater motor variability leads to a greater implicit adaptation driven by a large error. Moreover, the results suggest that error size affects this relationship. Error size may be an important factor in elucidating the effects of motor variability on motor learning.

## Figures and Tables

**Figure A1:**
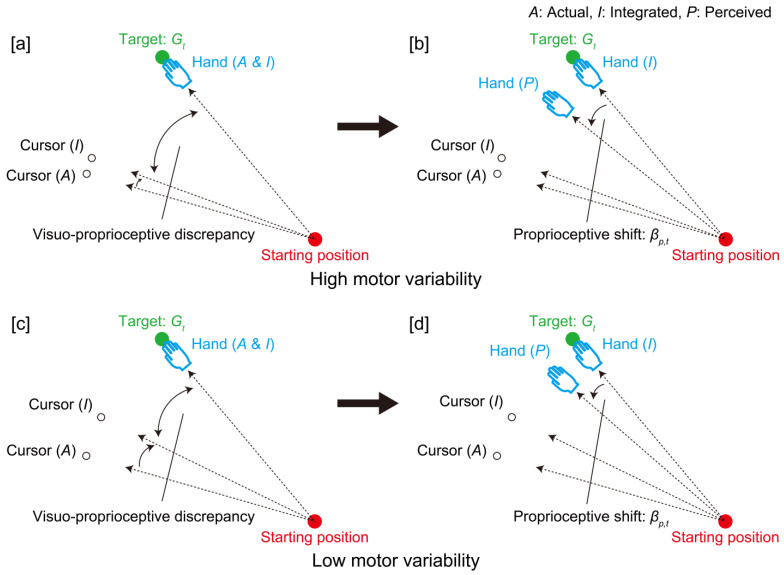
Effect of motor variability on trial-by-trial adaptation in the proprioceptive re-alignment model. (**a**,**b**): high motor variability. (**c**,**d**): low motor variability. This figure illustrates a trial in which the actual hand, Hand (*I*), could reach the target. Thus, the intramodal sensory recalibration is not driven in proprioception, and the actual hand and the integrated hand, Hand (*A*), are in the same position (**a**,**c**). Furthermore, this figure illustrates the case in which the magnitude of the proprioceptive shift is a fixed ratio of visuo-proprioceptive discrepancy (**b**,**d**). *A*, *I*, and *P* represent “actual”, “integrated”, and “perceived”, respectively; for example, Hand (*P*) refers to the perceived hand position.

## Data Availability

The data presented in this study are openly available in FigShare at https://doi.org/10.6084/m9.figshare.21665123.v2 (accessed on 3 March 2023).

## Supplementary Materials

The following supporting information can be downloaded at https://www.mdpi.com/article/10.3390/biology12030404/s1: Figure S1: Explanation of the experimental task in detail; Figure S2: Distribution of absolute hand angle in the baseline phase.

## Appendix A

The proprioceptive re-alignment model (PReMo) [32] first assumes the intramodal sensory recalibration in proprioception and vision. An optimal intramodal estimate of hand position is performed using the weighted average of the actual hand position ($x_{p,\;t}$) and the expected hand position based on an outgoing motor command ($G_{t}$) in trial t. Thus, the integrated hand position ($x_{p,\;t}^{I}$) is given:

(A1) $$x_{p,\;t}^{I}=\frac{\sigma_{u}^{2}}{\sigma_{u}^{2}+\sigma_{p}^{2}}x_{p,\;t}+\frac{\sigma_{p}^{2}}{\sigma_{u}^{2}+\sigma_{p}^{2}}G_{t},\;$$

where $\sigma_{u}^{2}$ and $\sigma_{p}^{2}$ are the uncertainty of sensory expectations/predictions given a motor command to the goal and the uncertainty in the proprioceptive system, respectively. Similarly, the integrated cursor position ($x_{v,\;t}^{I}$) is given by the weighted average of the actual cursor position ($x_{v,\;t}$) and the expected cursor position based on an outgoing motor command ($G_{t}$):

(A2) $$x_{v,\;t}^{I}=\frac{\sigma_{u}^{2}}{\sigma_{u}^{2}+\sigma_{v}^{2}}x_{v,\;t}+\frac{\sigma_{v}^{2}}{\sigma_{u}^{2}+\sigma_{v}^{2}}G_{t},\;$$

where $\sigma_{v}^{2}$ is the uncertainty in the visual system (Figure A1a,c). Subsequently, PReMo assumes the crossmodal sensory recalibration between proprioception and vision. The integrated hand position ($x_{p,\;t}^{I}$) shifts toward the integrated cursor position ($x_{v,\;t}^{I}$), resulting in the perceived hand position ($x_{p,\;t}^{per}$); the shift is referred to as a proprioceptive shift ($\beta_{p,\;t}$; Figure A1b,d):

(A3) $$x_{p,\;t}^{per}=x_{p,\;t}^{I}+\beta_{p,\;t}.$$

Regarding the rules governing the magnitude of the proprioceptive shift, PReMo assumes that while the magnitude is a fixed ratio ($\eta_{p}$) of visuo-proprioceptive discrepancy ($x_{v,\;t}^{I}-x_{p,\;t}^{I}$) for small discrepancy, the magnitude saturates ($\beta_{p,\;sat}$) for large discrepancy:

(A4) $$\beta_{p,\;t}=min\left(\;\left|\eta_{p}\left(x_{v,\;t}^{I}-x_{p,\;t}^{I}\right)\right|\;,\;\left|\beta_{p,\;sat}\right|\;\right).$$

The mismatch between the perceived hand position and its intended goal (i.e., proprioceptive error: $G_{t}-x_{p,\;t}^{per}$) is calculated; thus, the proprioceptive error drives trial-by-trial motor adaptation:

(A5) $$x_{p,\;t+1\;\;}=x_{p,\;t}+K\left(G_{t}-x_{p,\;t}^{per}\right),$$

where K is a learning rate.

In PReMo, it is assumed that high motor variability increases the uncertainty in sensory predictions ($\sigma_{u}^{2}$). According to Equations (A1) and (A2), such high variability can result in the actual position of the hand ($x_{p,\;t}$) or the cursor ($x_{v,\;t}$) having a relatively larger contribution to the integrated position of the hand ($x_{p,\;t}^{I}$) or the cursor ($x_{v,\;t}^{I}$). In this study, it was not certain whether the hand was positioned counterclockwise or clockwise relative to the target; but the cursor at the error-clamp trial with error always deviated from the target in either direction depending on the sign of the error type (±3°, ±6°, and ±12°). Therefore, via the optimal intramodal estimate of cursor position, high motor variability can increase the visuo-proprioceptive discrepancy ($x_{v,\;t}^{I}-x_{p,\;t}^{I}$) that drives the proprioceptive shift (Figure A1a,c). If the visuo-proprioceptive discrepancy is large enough that the proprioceptive shift saturates, the increase in visuo-proprioceptive discrepancy will not affect the proprioceptive shift; otherwise, the increase in the discrepancy will amplify the proprioceptive shift (Equation (A4); Figure A1b,d). In the latter case, high motor variability is expected to cause the large magnitude of trial-by-trial adaptation, as the amplification of the proprioceptive shift implies an increase in the proprioceptive error ($G_{t}-x_{p,\;t}^{per}$), which determines the magnitude of trial-by-trial adaptation (Equation (A5)). Note that the above predictions assume the trial-by-trial adaptation paradigm or an early adaptation stage when constantly presenting the same error-clamp feedback, rather than the late adaptation stage in which the hand is positioned quite far from the target in the direction opposite to that of the cursor.
