# Supplementary material for: Error Size Shape Relationships between Motor Variability and Implicit Motor Adaptation
Source: Biology (Basel). 2023 Mar 3;12(3):404. doi: 10.3390/biology12030404 (PMC10045141; doi:10.3390/biology12030404)
Supplement: Supplementary file 1 [file biology-12-00404-s001.zip › FigureS1.pdf]

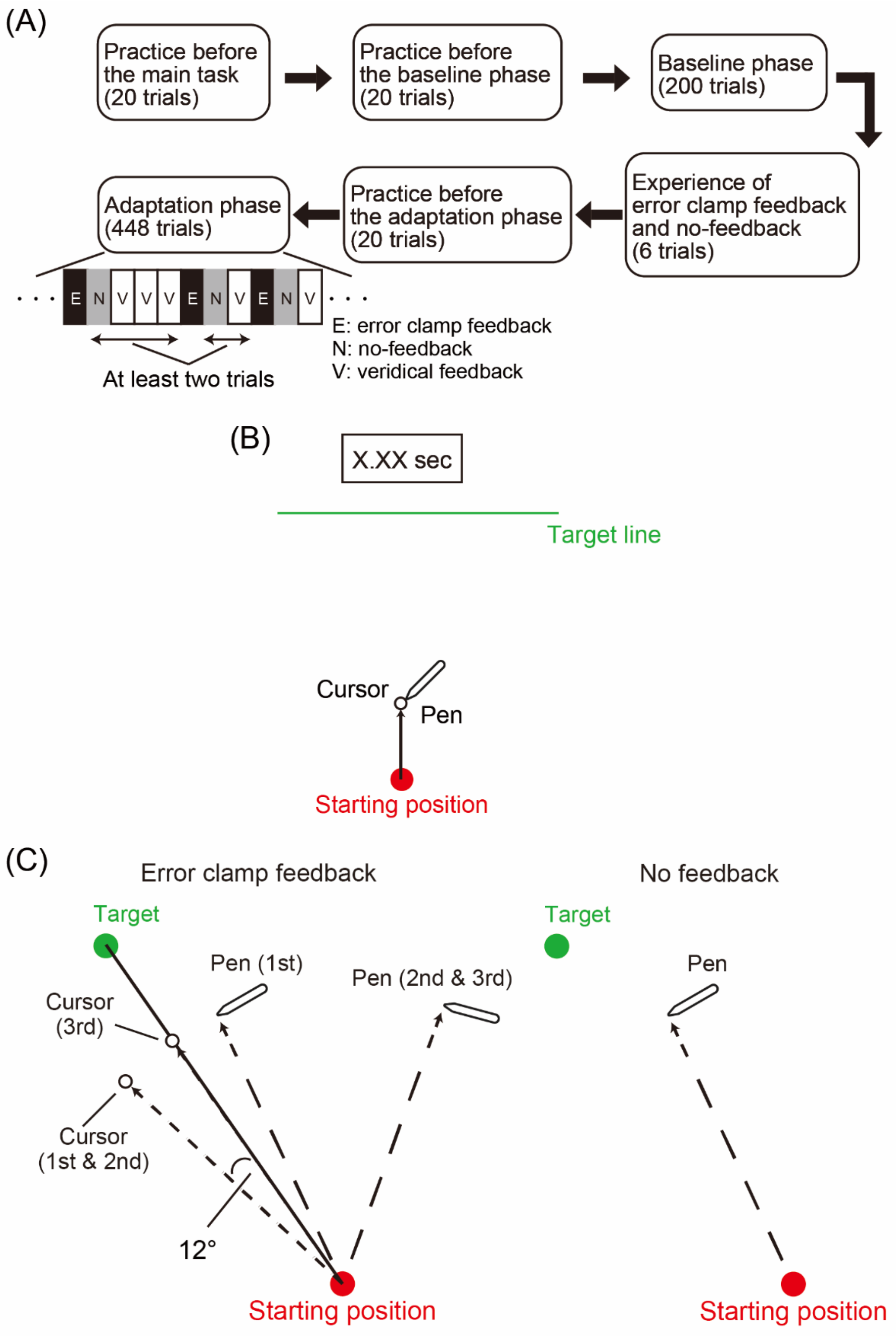

Figure S1. (A) Experiment schedule. Between each error clamp trial in the adaptation phase, there were at least two shooting movements under other trial types. (B) Practice before the main task. Participants performed the shooting movement to pass through a target line, positioned 10 cm away from the starting position at 90°. When the movement amplitude exceeded 10 cm, the cursor froze at the point and the movement time was displayed. They were instructed to move in 140 ms. (C) Experience of error clamp feedback and no-feedback. As the experience of error clamp feedback, in the first trial, participants performed the shooting movement toward the target and the cursor was deviated from the target by 12°; in the second trial, they moved toward the upper left, which was different from target direction and the cursor was deviated from the target by 12°; in the third trial, they moved toward the upper left direction and the cursor was deviated from the target by 0°. As the experience of no feedback, in each trial, participants performed the shooting movement toward the target and the cursor disappeared when the movement amplitude exceeded 1 cm.
