# Supplementary material for: Error Size Shape Relationships between Motor Variability and Implicit Motor Adaptation
Source: Biology (Basel). 2023 Mar 3;12(3):404. doi: 10.3390/biology12030404 (PMC10045141; doi:10.3390/biology12030404)
Supplement: Supplementary file 1 [file biology-12-00404-s001.zip › FigureS2.pdf]

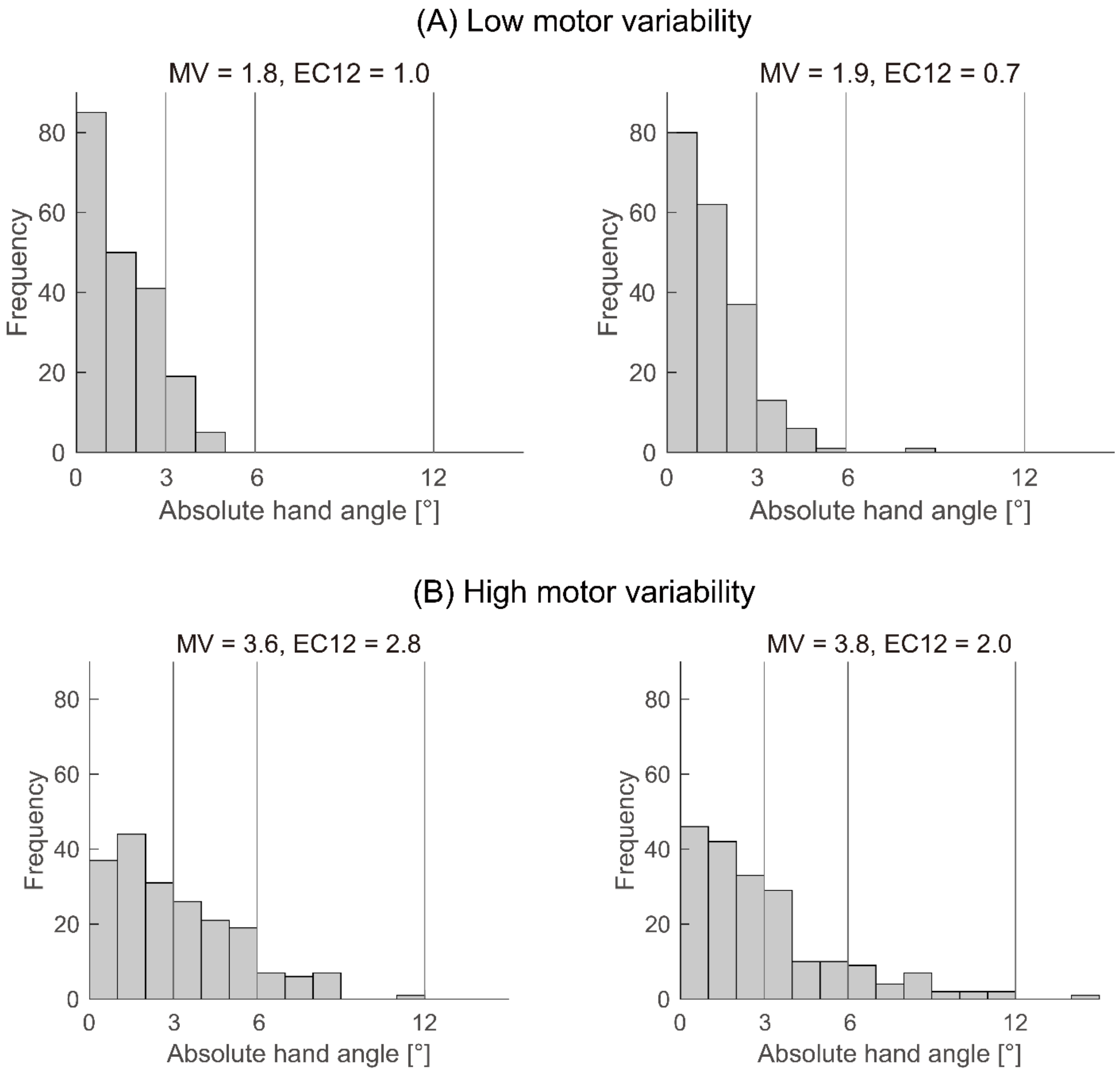

Figure S2. Absolute hand angle in the baseline phase. Representative participants with (A) low and (B) high motor variability. The histograms show that participants with high variability tend to have a wide distribution of the absolute hand angle in the baseline phase. MV and EC12 represent motor variability in the baseline phase and error correction for 12° absolute error, respectively.
